# Asymmetry of Cell Division in CFSE-Based Lymphocyte Proliferation Analysis

**DOI:** 10.3389/fimmu.2013.00264

**Published:** 2013-09-02

**Authors:** Gennady Bocharov, Tatyana Luzyanina, Jovana Cupovic, Burkhard Ludewig

**Affiliations:** ^1^Institute of Numerical Mathematics, Russian Academy of Sciences, Moscow, Russia; ^2^Institute of Mathematical Problems in Biology, Russian Academy of Sciences, Pushchino, Russia; ^3^Institute of Immunobiology, Kantonal Hospital St. Gallen, St. Gallen, Switzerland

**Keywords:** T cells, CFSE assay, asymmetric division, mathematical modeling

## Abstract

Flow cytometry-based analysis of lymphocyte division using carboxyfluorescein succinimidyl ester (CFSE) dye dilution permits acquisition of data describing cellular proliferation and differentiation. For example, CFSE histogram data enable quantitative insight into cellular turnover rates by applying mathematical models and parameter estimation techniques. Several mathematical models have been developed using different types of deterministic or stochastic approaches. However, analysis of CFSE proliferation assays is based on the premise that the label is halved in the two daughter cells. Importantly, asymmetry of protein distribution in lymphocyte division is a basic biological feature of cell division with the degree of the asymmetry depending on various factors. Here, we review the recent literature on asymmetric lymphocyte division and CFSE-based lymphocyte proliferation analysis. We suggest that division- and label-structured mathematical models describing CFSE-based cell proliferation should take into account asymmetry and time-lag in cell proliferation. Utilization of improved modeling algorithms will permit straightforward quantification of essential parameters describing the performance of activated lymphocytes.

## Introduction

The ability of the immune system to protect the host organism against live-threatening infections and tumors directly depends on the reactivity of lymphocytes to antigenic stimulation, with a key role of clonal T cell responses (1). The perception of infections as a race between the invading pathogen and immunity suggests that it is the knowledge of the proliferation and death rates of T cells which provides a quantitative basis for assessing the quality of the host immunity (2). For almost 20 years, flow cytometry-based analysis of intracellular fluorescent dye distribution has been used to assess the proliferative performance and differentiation patterns of lymphocytes (3–5). Since the prototype dye for this analysis is CFSE, the assay is commonly referred to as CFSE dilution assay or – more simply – CFSE assay. A quantitative characterization of T cell turnover which can be elaborated from time series of CFSE histograms ranges from “static” measures such as precursor cell frequency or mean generation number, to “dynamic” parameters characterizing the cell cycle progression and apoptosis rates (6). However, estimation of turnover parameters requires formulation of mathematical models of cell growth which can take various forms and differ in their complexity depending on the parameters of interest and the richness of the available data [comprehensively reviewed by De Boer and Perelson (7)]. Importantly, current approaches to the analysis of CFSE proliferation data are based on the assumption that cell division is symmetric, i.e., the fluorescent label is halved in the two daughter cells (3, 5, 7–9). However, a random and uneven partition of mass between the sister cells is considered as an axiom in cell biology since many years (10). Although the detailed knowledge of the intracellular reactions which affect the turnover and intracellular heterogeneity of CFSE labeled proteins is currently limited (11), it is broadly accepted that CFSE binds indiscriminately to intracellular proteins and the fluorescence intensity of any single cell is roughly proportional to the total number of CFSE molecules bound to proteins within that cell (12). The latter study proposed a method for the analysis of CFSE-labeling experiments which also considered the possibility of an unequal division of CFSE molecules between the daughter cells.

The inequality of the mass (protein) distribution to the daughter cells directly suggests that CFSE labeled proteins are unequally partitioned between daughter cells. Indeed, recent studies describing T cell activation showed that asymmetric cell division can be an inherent part of T cell growth and differentiation (13–16). However, direct experimental evidence for asymmetric partition of CFSE between daughter cells is still missing. Nevertheless, the existing deterministic mathematical frameworks should be amended to facilitate a quantitative analysis of CFSE-based lymphocyte proliferation when asymmetry of cell division associated with unequal partition of CFSE labeled proteins between the two daughter cell results in a poor resolution of divisional clusters in CFSE histograms. Here, we briefly summarize recent findings describing asymmetric lymphocyte division and progress in the analysis of CFSE-based lymphocyte activation. Moreover, cell proliferation is not an instantaneous process and it takes a finite time for a cell to progress from the G1-phase of the cell cycle to the completion of the M-phase. The duration of the continuous progression is called a time-lag and in general, needs to be explicitly parameterized in the model equations. Finally, we suggest that mathematical models describing CFSE-based lymphocyte proliferation should consider both asymmetry in division and time-lag in proliferation.

## Asymmetric Lymphocyte Division

Symmetric or asymmetric cell divisions refer to the mode of cell division which results in two phenotypically identical- or different-daughter cells, respectively. The phenotypic features could be the cell size, cell surface receptors, intracellular components such as proteins (including those labeled with CFSE), transcription factors, or messenger RNA (17). Hence, these phenotypic differences provide the basis for the functional differences in the daughter cells, i.e., their cell fates.

Following encounter with their antigen displayed in the context of major histocompatibility complex molecules, naïve T lymphocytes go through well-orchestrated series of divisions generating different populations of cells that fulfill immediate effector functions or generate long-lived immunological memory. Two basic models explain the generation of such functionally distinct T cell phenotypes. According to the “one naïve cell – one fate” model, naïve lymphocytes are instructed to generate either effector or memory progeny (18). In this model, instruction of T cells, for example, is achieved through interaction with professional APCs (19). Hence, to preserve the instructing signal(s) received during activation and to maintain equality of the cells throughout division, T cells should divide in a symmetric fashion. The alternative model proposes asymmetric cell division as the mechanism that allows naïve T cells to give rise to two different daughter cells. These are referred to as proximal or distal daughter cell depending on their proximity to the APC. Such asymmetric T cell division represents a process that allows single cells to give rise to two, phenotypically and functionally different daughter cells, and thereby permits diversification of cell populations. In other words, one of the daughter cells inherits the potential to differentiate into full effector cell (proximal daughter), while the second daughter maintains the stemness of the mother cell. This principle feature of asymmetric cell division has also been described in developmental studies examining neurogenesis (20). Likewise, adaptation of adult tissues to changing environmental conditions such as the content of the gut requires rapid adaptation of one cell fraction while other cells maintain their high proliferative potential (21).

The processes involved in activation and differentiation of T cells, for example during infection have to swiftly generate cells with direct effector function to efficiently restrict viral replication (1). At the same time, some T cells should retain their ability to proliferate in order to prevent exhaustion of certain T cell subsets (22) and to facilitate generation of long-lived memory T cells (23). Indeed, Chang et al. (13) demonstrated that division of CD8^+^ T cells specific for a viral peptide leads to the generation of daughter cells with different characteristics. CFSE-based assays revealed that asymmetry is established already during the first round of division and is dependent on the presence of the cognate antigen (13). Assessment of the protein content in the daughter cells generated during the first cell division showed that asymmetry established during mitosis is preserved throughout cytokinesis. Moreover, proximal and distal daughter cells exhibit different protein expression profiles and functional properties with proximal daughter cells exhibiting higher immediate protective capacity (13). The finding that proximal daughter cells exhibit higher CD8 co-receptor and LFA-1 expression facilitating formation of more frequent and longer lasting interactions with antigen presenting APCs (14) further emphasized that asymmetric division critically determines both T cell phenotype and function.

Asymmetric cell division is not only an important feature of CD8^+^ T cell activation (13, 14), but also occurs during the activation and differentiation of CD4^+^ T cells (13, 24) and B cells (25, 26). While naïve CD8^+^ T cells require only one or only few encounters with APCs to proliferate and differentiate into effector cells, naïve CD4^+^ T cells depend on multiple encounters in order to differentiate and to exhibit specialized effector functions (27). Hence, it is likely that CD4^+^ T cells acquire their distinct phenotypes, e.g., Th1, Th2, or Th17, through multiple sequential asymmetric cell divisions. However, recent studies suggest that asymmetric cell division cannot be considered as the only mechanism that leads to the profound heterogeneity of T cell lineages (16). Thus, more research is required to resolve the contribution of sequential asymmetric T cell division to the generation of diverse T cell phenotypes. We suggest that a combination of CFSE-based T cell proliferation analysis with mathematical modeling may help – at least in part – to clarify this issue.

## Current Mathematical Models for CFSE-Based Lymphocyte Proliferation Analysis

**Figure 1:**
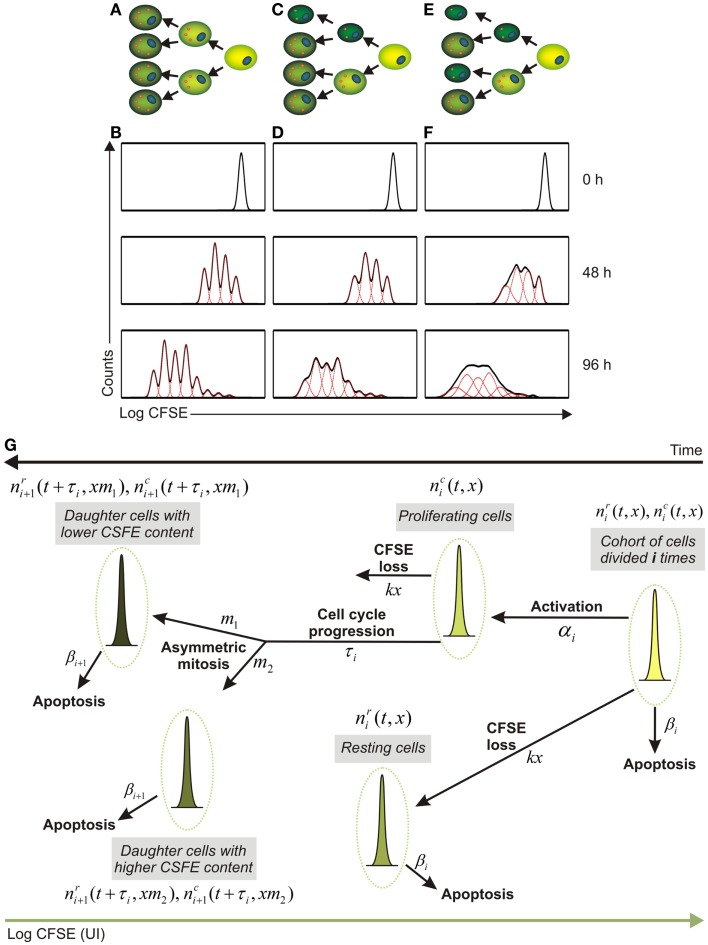
**Impact of asymmetry in T cell division impinges on fluorescent protein partition between daughter cells**. **(A,B)** Symmetric cell division with equal distribution of the fluorescent dye between daughter cells **(A)** and modeled time course analysis of T cell proliferation as determined by flow cytometry [**(B)**, solid black lines]. Dashed red lines in **(B)** indicate the evolution of CFSE intensity of the cohorts (generations) of cell which differ in the number of completed divisions with the assumption of symmetric division. **(C,D)** Asymmetric cell division with “low” asymmetry **(C)** and modeled flow cytometric time course analysis of CFSE dilution [**(D)**, solid black lines] that corresponds to an asymmetry 46/54% [**(D)**, dashed red lines describe the CFSE distributions for cell cohorts differing in terms of the completed divisions]. **(E,F)** T cells dividing with “high” asymmetry **(E)** and corresponding model-generated flow cytometric CFSE dilution patterns [**(F)**, solid black lines] with asymmetry values of 42/58% describing the behavior of the T cells in this setting [**(F)**, dashed red lines describe the cell cohorts corresponding to different generations]. **(G)** Schematic representation of the structure of a mathematical approach which considers the division- and CFSE label-heterogeneity of proliferating cells as well as asymmetry and time of cell division. Some cells from the cohort of cells which completed “*i*” divisions are activated (α*_i_* characterizes the activation rate) and progress through the cell cycle (τ*_i_* stands for the duration of the progression through S-G_2_-M phases), resulting to the generation of daughter cells which differ with respect to their CFSE content. Asymmetric mitosis refers to cell division which results into appearance of two phenotypically different daughter cells with a smaller and larger cell mass, respectively. These cells are characterized by an unequal amount of CFSE labeled proteins (*m*_1_ and *m*_2_ = 1−*m*_1_, describe the fractions of CFSE from the mother cell inherited by the two daughter cells). The natural decay of the CFSE fluorescence intensity is taken into account (*kx* – stands for an exponential decay of CFSE loss).

Several mathematical models have been established for the analysis of CFSE-based proliferation assays (7, 9, 12, 28–32). The existing modeling frameworks can be subdivided on the basis of the major requirements for CFSE histogram data processing into two main categories (Table 1). The first group requires a decomposition of the CFSE histograms characterizing the distribution of cells with respect to the fluorescent dye into the distinct generations of cells. The procedure is based on fitting the CFSE histogram with a series of log-normal Gaussian distributions differing in their means and standard deviation and is implemented in commercially available standard software packages. Importantly, the assignment of distinct cell generations to CFSE clusters has remained an empiric process which depends heavily on initial labeling homogeneity, label degradation, cellular auto-fluorescence, and other factors including experimental skills of the researcher (33). As long as the division is symmetric (or almost symmetric) (Figure 1A), these factors can be tuned in a proper way to enable resolution of successive generations as distinct CFSE clusters (Figure 1B). Under these conditions a range of existing mathematical models can be tuned to estimate the turnover parameters of the stimulated lymphocyte population. The key features of the corresponding families of the models are outlined in Table 1, rows one to three. These models describe the population dynamics of cells which differ in the number of completed divisions and ignore the heterogeneity of the cells within a generation with respect to the CFSE content. The immunologically relevant issues that were addressed with the models of this group include regulatory effects of IL-2 on the T cell responses (34, 35), regulation of hematopoietic stem cells cycling (36), and kinetics of mouse erythroid progenitor cell differentiation (37).

**Table 1:** **Major features of mathematical models describing CFSE-based proliferation assays**.

Cell proliferation model^1^	Input data	Estimated parameters	Mathematical approach^2^	Primary sources
A–B state model	Generation structure	Division entry-, apoptosis- rates, duration of division	DDE	Nordon et al. (6), Ganusov et al. (28)
G_0_ model	Generation structure	Division entry-, apoptosis rates, duration of division	hPDE	Bernard et al. (51)
Random birth-death	Generation structure	Division-, apoptosis rates, progressor fraction	ODE, IE, branching processes	Ganusov et al. (28), Yates et al. (29), Lee et al. (30), Zilman et al. (31), Hyrien et al. (41), Veiga-Fernandes et al. (52), Revy et al. (53), Hawkins et al. (54)
Random birth-death, CFSE-structured	CFSE histograms	Division-, apoptosis-, CFSE decay rates	hPDE	Luzyanina et al. (38)
Random birth-death, generation-, CFSE-structured	CFSE histograms	Division-, apoptosis-, CFSE decay rates, auto-fluorescence	hPDE	Hasenauer et al. (40), Banks et al. (32)
Asymmetric division, G_0_-model, generation-, CFSE-structured	CFSE histograms	Asymmetry, division-, apoptosis-, CFSE decay rates, time-lag of proliferation	hPDE	See text for details

*^1^The following notations are used: “A–B state model” refers to the model of cell cycle in (55) in which the intermitotic period is composed of an A-state (major part of G1-phase) and a B-phase (conventional S, G2, and M phases); “G0 model” refers to the view of the cell cycle with two states (47), i.e., resting- (G0) and cycling- states (G1, S, G2, M). Conceptually, it is equivalent to the A–B state model. “Random birth-death” model refers to a discrete compartmental (generation structured) model of cycling cells (56). “Generation structured” refers to the mathematical model in which the cell population is decomposed into cohorts of cells which differ with respect to the number of completed division cycles; “CFSE-structured” represents the mathematical description of cell population in which the distribution (heterogeneity) of cells with respect to the fluorescence intensity is followed by considering the cell distribution function*.

*^2^DDE, delay differential equations; hPDE, hyperbolic partial differential equations; ODE, ordinary differential equations; IE, integral equations*.

The second group of models which refer directly to the CFSE histograms seems to be more appropriate when the generational structure of the labeled population cannot be easily resolved (Table 1, rows four to five). The initially proposed model describes the evolution of the labeled cells distribution with respect to the CFSE level (38). Although this and similar models proved to be functional in estimating the proliferation- and death rates as functions of the structure variable directly from the histogram data (38, 39), the problem of translating the estimated functions into biologically meaningful parameters still requires the knowledge of the division structure of the lymphocyte population. A major breakthrough in the improvement of the distributed parameter models for the dynamics of heterogeneous CFSE labeled cell populations were recently proposed division- and label-structured mathematical models (32, 40). The major potential of this framework as an analytical tool is based upon the following features: (i) no need for CFSE histogram decomposition, (ii) characterization of cell growth in terms of generation dependent division- and death rates, (iii) an explicit form of the dependence of solution on the turnover parameters.

Another class of recently developed mathematical models which allow a direct fitting of the CFSE histograms is based on branching processes (12, 41). The approach allows for probabilistic characterization of cell activation, proliferation, and death from the CFSE dilution data and does not require the assumption about equality of CFSE division between the two daughter cells.

The first and the second group of models rely on the premise of symmetric cell division. However, tracing proliferation of other cell types such as cancer cells has been reported to be difficult (42) due to poorly resolved peaks of the different cell generations. Since cytokinesis is not perfect, it was suggested that the two daughter cells are unlikely to inherit exactly half of the CFSE fluorescence dye of the mother cell. An increase in the degree of the asymmetry of mass partition between daughter cells and hence disparate distribution of fluorescently labeled proteins should result in a poorer resolution of generational clusters as shown in Figures 1C,D for lower asymmetry and in Figures 1E,F for higher asymmetry. This in turn will lead to the generations overlap in CFSE histograms thus posing a limit to experimentalists’ ability to resolve the individual generations using conventional decomposition methods.

## Modeling Asymmetric Division of CFSE Labeled T Cells

We have been recently dealing with the analysis of the proliferative performance of monoclonal CD8^+^ T cells recognizing an H2-Kb-binding epitope derived from the S protein of the mouse hepatitis virus (MHV). Clearance of MHV during acute infection is achieved through the combined action of type I interferons (43) and CD8^+^ T cells (44). Moreover, CD8^+^ T cells essentially contribute to control of the virus during persistent infection, for example in the central nervous system (45). We have initiated a project on the generation of avidity-tuned, antigen-specific T cells for adoptive transfer as an option to augment antiviral immune responses during chronic infection. To this end, MHV-specific T cell receptors (TCRs) were cloned and tested in retrogenic systems (46). *In vitro* re-stimulation of the CFSE labeled monoclonal CD8^+^ T cells showed that CFSE dilution was characterized by broadly varying patterns from highly distinct peaks to poorly resolved generational clusters. We propose that an explicit consideration of the asymmetry in protein partition between the daughter cells facilitates a consistent mathematical description of CFSE histogram time series data (Figure 1G). The appropriate mathematical framework should describe the population of CFSE labeled T cells by the distribution of cells with respect to CFSE amount (unit of intensity, UI). The subpopulations differ in terms of completed rounds of division and are further distinguished in resting and proliferating states, with the respective notation and *i* standing for the generation (number of completed divisions), *t* – for time and *x* – for CFSE amount per cell. A conceptual scheme of the modeling approach is shown in Figure 1G suggesting that such a model can be naturally formulated as an extension of a generation- and division-structured population balance model with the cell cycle represented according to the G_0_ model (47) and the division asymmetry explicitly taken into account.

Under conditions of symmetric CD8^+^ T cell division with the difference of protein partition between the sister cells being equal to zero (i.e., every daughter cell inherits half of the fluorescently labeled proteins of the mother cell), the model should predict clearly distinct generations (Figure 1B, dashed red lines). If the division is “weakly” asymmetric, i.e., the protein partition between the sister cells is different, the width of the CFSE distribution of the successive generations should become broader (Figure 1D, dashed red lines). Further increase in the degree of the asymmetry would result in a substantial overlap of the distinct cell generations (Figure 1F, dashed red lines). Obviously, this type of behavior of T cells – and other cells such as tumor cells needs to be regarded as a cause of a poor resolution of the generations in CFSE histograms (Figures 1D,F, solid black lines) thus creating an obstacle on the application of standard CFSE analysis tools.

The fitting of mathematical models for asymmetric cell division as conceptualized in Figure 1G to the time series data provides a tool for the estimation of the cell physiology parameters such as: (i) the generation-specific activation and death rates (α*_i_*, β*_i_*); (ii) the duration of the division cycle characterized by the time-lag (τ*_i_*); (iii) the division asymmetry factors (*m*_1_ + *m*_2_ = 1), specifying the fraction of proteins which is inherited by the first and the second daughter cells, respectively; and (iv) the natural decay of the CFSE fluorescence intensity of the labeled cells (parameterized as *kx*). Taken together, asymmetric cell division improves assessment of T cell performance parameters from CFSE-based proliferation assays, even under conditions of poorly separated peaks.

## Concluding Remarks

It is considered that the regulation of cell expansion and differentiation can occur by modulating the degree of asymmetry of cell divisions (17). It has been clearly shown that T lymphocyte division in response to pathogen exhibits unequal partitioning of proteins that mediate signaling and cell fate determination (13). Hence, asymmetric T lymphocyte division provides an additional mechanism for generating functionally heterogeneous populations of CD8^+^ T cells both in primary and memory adaptive immune responses (48). Since a precise mechanistic link between the quantitative differences in partitioning of specific proteins between daughter cells and the developmental path of antigen-specific T cells remains to be established (49), mathematical modeling is now a key “instrument” for understanding the regulation of individual cell fates (15, 16, 50).

The addition of asymmetric T cell division to the analysis of CFSE-based proliferation data fills important gaps as it: (i) allows one to estimate the proliferation parameters for asymmetrically dividing cells directly from CFSE histograms with poorly resolved generations peaks and (ii) introduces a quantitative parameter which characterizes the difference in the partition of the fluorescent proteins between daughter cells and can be directly estimated from the same CFSE dilution data. A further question in CFSE analyses open for examination is the interplay between experimental variability, biological variability, and model parsimony. We expect that new mathematical tools for the analysis of a fundamental property of cell division, i.e., the phenotypic identity or differences of the daughter cells known as asymmetry, will be developed and introduced into daily experimental work. Thereby, a better understanding of the diversity and mechanisms underlying activation and homeostasis of T cell responses will be achieved.

## Conflict of Interest Statement

The authors declare that the research was conducted in the absence of any commercial or financial relationships that could be construed as a potential conflict of interest.
